# The rise of large-scale imaging studies in psychiatry

**DOI:** 10.1186/2047-217X-3-29

**Published:** 2014-11-25

**Authors:** Jessica A Turner

**Affiliations:** Department of Psychology and Neuroscience Institute, Georgia State University, P.O .Box 5010, Atlanta, GA 30302 USA

**Keywords:** Neuroinformatics, Data sharing, Clinical imaging, Data mining

## Abstract

From the initial arguments over whether 12 to 20 subjects were sufficient for an fMRI study, sample sizes in psychiatric neuroimaging studies have expanded into the tens of thousands. These large-scale imaging studies fall into several categories, each of which has specific advantages and challenges. The different study types can be grouped based on their level of control: meta-analyses, at one extreme of the spectrum, control nothing about the imaging protocol or subject selection criteria in the datasets they include, On the other hand, planned multi-site mega studies pour intense efforts into strictly having the same protocols. However, there are several other combinations possible, each of which is best used to address certain questions. The growing investment of all these studies is delivering on the promises of neuroimaging for psychiatry, and holds incredible potential for impact at the level of the individual patient. However, to realize this potential requires both standardized data-sharing efforts, so that there is more staying power in the datasets for re-use and new applications, as well as training the next generation of neuropsychiatric researchers in “Big Data” techniques in addition to traditional experimental methods. The increased access to thousands of datasets along with the needed informatics demands a new emphasis on integrative scientific methods.

## Review

The history of Magnetic Resonance Imaging (MRI) studies in biomedical and psychological research is one of increasingly widespread and sophisticated applications. From initial publications of a single or handful of subjects, a classic paper [1] argued that at least 12 subjects were needed to identify an effect in functional MRI data; indeed, analyses with fewer than 20 subjects are still common, (e.g., [2]). But in recent years, studies 600 or more scanning samples collected on a single scanner are appearing (e.g., [3]). The Enhancing Neuroimaging Genetics through Meta-Analysis (ENIGMA) meta-analysis approach used data from over 10,000 individuals, pulling from multiple legacy datasets and scanners [4]. There are a number of approaches to large-scale neuroimaging studies; they are not interchangeable, as they have complementary strengths and weaknesses. However, the growing tendency toward large-scale studies and data analysis brings with it certain calls to action for the field of neuroimaging in clinical research as it moves into the realm of recognizable “Big Data” [5].

### Why has large-scale imaging come about?

The statistical mantra is that more subjects means more power; and how many subjects are needed depends, of course, at least in part on the effect size of the question under study. To ascertain where in the brain functional MRI (fMRI) signal changes are related to different conditions in a simple cognitive task, 10–15 subjects may be sufficient; studies of the neural correlates of auditory hallucinations in psychotic populations have largely pulled from smaller samples of 1–10 [6], though larger studies of 15–30 have been painstakingly collected [7, 8]. The Functional Imaging Biomedical Informatics Research Network (FBIRN), in one of the first “multi-site” fMRI studies, collected 200–300 patients with schizophrenia and controls using in the same fMRI protocol across multiple universities, motivated in part by inconsistencies found in smaller samples regarding frontal cortex function in fMRI studies of schizophrenia [9, 10]. Larger-scale neuroimaging studies are also motivated on occasion by a desire to expand the clinical picture of the sample, providing a larger variability in symptom profiles, for comparisons of clinical variation within a single-diagnosis sample [11] or for longitudinal prognosis predictions in the face of high individual variation [12].

A similar sample size of several hundred is often needed for the most basic analyses of genetic effects on imaging measures, such as testing the relationship between variation at a single genetic locus and the BOLD signal during working memory [13, 14], or to identify the combined effects of selected multiple genes on brain structural variation [15]. However, genetic effects are notoriously small and unreliable; to examine neuroimaging effects of the entire genome rather than targeted subsets of genes, data from tens of thousands of subjects are required [4]. These latter are truly large-scale studies.

### Categories of large-scale imaging studies

There are some useful categories of design for imaging datasets of 100 or more subjects, considering the level of control and planning that is used. The most controlled are the planned, coordinated and often multi-site imaging studies. Less controlled are the Aggregated Mega-analyses, in which existing, often legacy datasets with similar imaging techniques and sample populations are combined for analysis. The next is Opportunistic studies, which are often seen at institutions or combinations of institutions that make their imaging data available for mining, without regard for similar sample populations or imaging protocols. Historically, the most common method, that does not control the collection of imaging data or aggregate it in one place at all, are Meta-analyses, which can be either *ad hoc* or *prospective*. We consider each of these in turn.

#### Planned studies

Planned studies can be large scale while being collected at a single site, using a consistent protocol for both subject recruitment and data collection. Examples of this can range from several hundred cases vs. controls [16, 17], to the Genetics of Brain Structure (GOBS) dataset of encompassing more than 1,000 subjects from a multi-pedigree study of heart disease [18], or the Philadelphia Neurodevelopmental Cohort with 1,445 imaging datasets on a single scanner [19]. In the last 15 years, the ability and the will to collect structural, functional, diffusion and perfusion imaging data across multiple imaging centers has developed, with FBIRN collecting several hundred patients with schizophrenia and controls across eight centers in the United States [10, 11], the Multisite Clinical Imaging Consortium (MCIC) doing the same across four centers [20], the Alzheimer’s Disease Neuroimaging Initiative (ADNI) collecting 800 subjects longitudinally over 50 centers [21, 22], and the PREDICT-HD data collecting over 1,400 subjects longitudinally for ten years across 32 imaging centers, even more impressive given the rarity of Huntington’s Disease [23]. This phenomenon is by no means limited to the US, of course; the Thematically Organized Psychosis (TOP) study in Norway collected over 600 imaging datasets on patients with schizophrenia, bipolar disorder and controls [24]; the IMAGEN study is an international and longitudinal imaging and genetics study of mental health in adolescents, with several thousand subjects participating [25]. These are merely examples, not an exhaustive list.

There are pros and cons to these studies, of course. Notably, these studies are very expensive. In a multi-site study, effort and expense is not simply a linear sum of doing the same but smaller study at each site; the coordination, planning, and equilibration of methods and equipment across sites [26], the infrastructure for moving data to central locations for analysis, and the time involved in keeping everyone up to date on any changes in the protocols, forms a necessary and costly overhead for these tightly organized studies. However, there is as good a guarantee as one can get in the real world that these samples are comparable across sites. Sources of variance have been minimized as much as possible. The subjects are recruited using the same criteria, the scanners are calibrated to the same levels, the protocols are identical wherever possible, the data are analyzed using the same quality assurance methods and software [27]. Just as clinical trials for FDA approval are controlled and prescribed, these kinds of studies are, effectively, FDA clinical trials methods translated as closely as possible to imaging studies--and the investment by the funding source is similarly demanding.

#### Aggregated mega-analyses

Aggregated Mega-analyses are studies that combine existing datasets without prior coordination. They are commonly limited to a single imaging modality, for example T1-weighted structural images or resting state fMRI, without requiring that the imaging parameters be the same across datasets. They may be limited to a particular clinical population, or that the data include a particular set of clinical assessments, without requiring that all subjects have been recruited the same way or that the same diagnostic criteria are rigidly applied. The confusion between schizophrenia and schizoaffective diagnoses is a standard example: some investigators combine both diagnoses in their samples, while others keep them separate. In a planned multi-site study, that point would be standardized across subjects; in an aggregated mega-analysis, one is often stuck with the ambiguity.

A notable example of an aggregated dataset and mega-analysis is the 1,000 Functional Connectomes project [28], which collated 1,414 subjects’ resting state fMRI data across 35 imaging centers worldwide, without regard to imaging protocol. The only constraints they set were that subjects be healthy controls over 18 and under 60 years old. They identified an underlying, robust infrastructure of resting-state signals across the brain that has been the canonical result since their publication. They were able to set the foundation for the effects of age in that range, gender, and the similarity of results across different analysis methods for the resting state brain in healthy subjects.

The advantage to this approach is clearly the ability to collate large datasets fairly cheaply, from investigators who are willing to share. The Autism Brain Imaging Data Exchange (ABIDE) dataset of autism imaging [29], for example, includes resting state fMRI data from over 1,000 participants aggregated across 16 different sites, and since its release in 2012, has resulted in six published papers on the aggregated set, with many more in preparation. In schizophrenia studies, Cota *et al.* (under review) [30, 31] has aggregated structural imaging data from over 1,800 subjects from eight legacy studies to evaluate gray matter loss. The recently released Consortium for Reliability and Reproducibility (CORR) dataset [32] collated structural and functional imaging data from over 1,600 subjects, available to the community. All of these are imaging and related data that have been already collected through other funding sources; the cost involved for the aggregations is mostly the personnel and time needed to send and receive the datasets for analysis, process and curate them, and the analysis time and effort. While the curation process can be lengthy, time-consuming and frustrating, it pales in comparison to the original subject recruitment and scanning costs for large scale studies.

The challenges for this method are primarily the increased variability in the images, since the imaging protocols vary widely. As noted extensively by Glover *et al.*[26], changes in scanning parameters can create protocol-specific deformations in the image, as well as changes in the relative contrast between tissues, and thus affect estimates of any brain measure being used, whether functional or structural. The papers cited so far on aggregated mega-analyses deal with inter-site variation in a number of ways, often through modeling site as a covariate or factor in the statistical model. However, the loss of sensitivity through increased variability has to be weighed against the increased in generalizability. More subtle effects may be lost, but those that remain are more robust.

The differences in sample characteristics are also a challenge; the sample sizes drop immediately as soon as more is required than the image and some basic demographic information. Studies were conducted with different clinical and cognitive assessments, which are generally not comparable. The advantage of power in the large sample sizes is then lost when more nuanced questions need to be asked about duration of illness, the role of cognitive deficits, or aspects of the subjects’ medical history, and the data simply aren’t there.

#### Opportunistic studies

Opportunistic studies refer to the growing practice of scanning centers creating institutional data repositories. The Mind Research Network in Albuquerque (NM, USA), has a policy that all scans performed on its scanner are part of its data repository for controlled sharing [33]. The four MRI scanners at the Donders Institute for Cognitive Neuroscience have provided structural imaging data for the Brain Imaging Genetics (BIG) study, from the pool of images from all college students being scanned for many other research projects [34]. The University of California, Irvine (UCI), and the University of Southern California (USC) have agreed to develop a repository of non-emergency MRI scans from both institutions [35]. The studies that come from these sorts of repositories are opportunistic in the sense that the subjects are whoever was scanned for other studies, and the imaging protocols are whatever was used for that study. In certain cases, such as the federated repositories from the Mind Research Network, and the UCI/USC network, a standardized if minimal imaging protocol can be agreed on, so that all non-emergency subjects receive the same structural and diffusion tensor imaging or resting state functional imaging protocol.

The effort behind these institutional-level data sharing methods can be extensive, requiring high-level administrative involvement, support, and assurances to develop a system for managing all the imaging data collected at an institution, as well as intrusion into the individual investigator’s methods, adding verbiage about data sharing to the protocol and consent forms, and limiting perhaps the scanning protocols that can be used. However, the repositories that result from it can be immense. The One Mind for Research project is leveraging these sorts of efforts, with the goal of collating datasets from several thousand traumatic brain injury (TBI) subjects from participating trauma centers and emergency room locations, as well as developing a registry over time of 25,000 patients seen for a suspected TBI and their computerized tomography (CT) scans [36].

While these approaches in many cases share the disadvantages of aggregated analyses—varied imaging protocols in some cases, incomplete clinical pictures in others--examples of the findings resulting from these efforts demonstrate their value. In a paper by Allen *et al.*[3], resting state fMRI datasets from over 600 healthy controls ranging in age from 12 to 71 years were pooled for an extensive and foundational study of the effect of age and gender on resting state networks and their interconnectedness. While this is not the size of the Functional Connectomes sample, it has the advantage of being collected using a standardized imaging protocol. In contrast, the aggregated BIG sample of 1,400 healthy controls is not standardized, but access to the original imaging data from a single institution allowed an in-depth analysis of the widely ranging imaging parameters on various gray matter measures [34].

#### Meta-analysis

Meta-analyses of published neuroimaging studies are important for developing consensus in the field. Standard meta-analyses combine results across smaller studies, identifying where the weight of the evidence falls in the case of conflicting results. These are *post hoc* meta-analyses, extracting published results and effect sizes from the literature; given the unknown number of unpublished analyses, these methods must account for the “desk drawer” phenomenon by various means. A particularly fruitful approach was developed by Laird and colleagues [37, 38], which leveraged the standardized systems for reporting fMRI results as coordinates in a three-dimensional brain space. The ability to statistically combine these coordinate-based analyses across studies has since resulted in over 400 publications with meta-analyses in schizophrenia, anxiety disorder, executive function, and many more topics [39]. Meta-analytic techniques have been applied to structural and task-based functional imaging; the rising popularity of resting state fMRI with its numerous analytical techniques [2, 3, 29, 40, 41], particularly multivariate ones, provides a particular challenge for *post hoc* meta-analyses. Overall, however, the advantages of *post-hoc* meta-analyses are well known, as are the disadvantages so these are not reviewed here.

A different approach is a *prospective* meta-analysis (along the lines of [42]), in which the results are not chosen from the published literature. Rather, legacy datasets are analyzed individually using a standardized statistical model, and the individual results are then pooled as in a usual meta-analysis. The largest project of this sort in neuroimaging to date is the ENIGMA project, which successfully collated statistical results from planned, consistent analyses of 10,000 subjects from 17 studies worldwide to identify the genetic effect on hippocampal volume and overall brain size [4]. The ENIGMA technique asked researchers to segment their structural imaging data into various brain region volumes using a standardized protocol (in one of two well-known software systems, Freesurfer or FSL [43, 44]), perform a standardized quality assurance protocol to remove bad data, and then they leveraged the standardized outputs from those software systems to develop scripts or programs in R [45], which would run over an entire dataset with minimal input from the dataset owner. Other than imaging data quality, image processing steps and analysis, there was very little control. The subjects could be anybody—the studies included patients with schizophrenia, attention deficit depression, autism, as well as “controls”—though they were required to be genetically Caucasian for analysis of genetic effects on the brain volumes. And notably, in this approach the data are not shared or centralized for analysis. The analysis techniques for each dataset are standardized, and the results from each dataset are what is shared. The meta-analysis is then performed on the effect sizes from each dataset.

This “crowd sourcing” approach to imaging genetics has continued successfully [46]. The ENIGMA project now has a number of collaborative working groups varying in size, exploring these same issues in distinct neuropsychiatric disorders [47]. There are papers in development on prospective meta-analyses of structural brain measures in schizophrenia, attention deficit syndrome, major depressive disorder, and bipolar disorder, with the combined expertise of hundreds of professionals in these fields.

Like the other uncontrolled designs, the prospective meta-analysis approach can be hampered by the variability in the collected data. Currently there are no standard batteries of clinical, cognitive, and socioeconomic measures that are applied to all imaging studies of schizophrenia; for example, individual studies are designed to answer specific questions, and collect the relevant data for their hypotheses. One dataset may include an extensive cognitive battery, while another does not include even basic IQ measures. Another dataset may have an equally extensive cognitive battery, but not the same one, leading to its own issues in comparability of measures. Like the mega-analysis or opportunistic study designs, these meta-analyses can end up with a “lowest common denominator” approach, including only basic covariates such as age and gender, unable to count on basic information about the duration of illness or medications being comparable or even available across the datasets.

The cost of a crowd-sourced approach, such as the ENIGMA model is in unpaid labor in many cases. ENIGMA and its subprojects are not planned multi-site studies, with staff at every site funded to work on their part of the analyses. They are almost entirely a volunteer army, of researchers willing to participate because it is a good experience, it is a project that can not be completed any other way, they believe in data sharing and aggregation, and are willing to leverage other funding sources to make it happen. While that may change in the future, the current model (as of the summer 2014) includes largely donated time and resources. That may not be an approach that supports growth in the long term, though the current level of energy for these projects from around the world is notable.

Some of the differences across study designs have been summarized in Table 1. These level descriptions are somewhat arbitrary; within any given category there will be some studies that are easier and some that are harder to perform, for example, based on the particular design and requirements.

**Table 1:** **Comparison of study categories**

Category	Comparability across datasets or sources	Control over analyses ^1^	Depth of phenotyping available	Ease of collecting a large sample ^2^
**Planned studies**	Highest possible similarities in data	Highest	Can be very deep but specific to the study hypotheses	Most difficult
**Aggregated mega-analyses**	Moderate; Some filtering of available datasets for improves comparability	High	Low to Moderate	Moderately easy; it requires existing datasets and investigators willing to share them
**Opportunistic studies**	Moderate, but can be high if scanning centers agree to use minimal standard protocols	High	Moderate	Moderately difficult; it often requires institutional arrangements to get started
**Meta-analyses (post-hoc)**	Lowest	Lowest	Lowest	Easiest; all published results that fit the meta-analytic question are available
**Meta-analyses (prospective)**	Lowest	High	Lowest	Moderate; it requires existing datasets and teams to analyze them in collaboration

^1^: *Control over analyses* covers whether planned analyses across each subset of the dataset are done the same way, either by a central authority or planned and agreed upon across groups performing the analyses.

^2^: *Ease* refers in a general way to the efforts involved in design and calibration, data aggregation and analysis, apart from study specific issues of the particular clinical population or obtaining any necessary funding, for example.

### The rise of large-scale studies leads to big data methods in neuroimaging

The goal of large-scale clinical neuroimaging is often the largest sample size available. Datasets from multiple research centers, multiple cities, and various countries are more likely to capture the range and variance of the clinical population than are smaller samples from a single center. Given that neuroimaging studies often pull from a limited sample of the population to begin with—subjects who are capable of undergoing neuroimaging—the more representative the sample can be, the better. All of these methods of large-scale data collection are geared toward this end, whether the goal is a genetically well-powered sample or simply capturing enough of the clinical variation. The studies presented, as examples above, have all been markedly successful in achieving these ends.

All the study designs reviewed here allow both replication and discovery. It is not only the planned studies which can test hypotheses; it is not only the less controlled categories of studies which support exploratory analyses. The ABIDE dataset, for example, while the result of aggregating legacy data, has been used to explore specific hypotheses regarding the relationship between functional connectivity of the posterior temporal sulcus and emotion recognition in autism [48]. The FBIRN III study protocol, in contrast, was designed primarily to examine the interaction between emotional distraction and working memory encoding in schizophrenia, with resting state data as an extra scan; however, the resting state data has resulted already in four papers published or under review, with more in preparation, exploring the relationships between various imaging features and disease state or clinical measure [40, 41, 49, 50]. The ADNI and COBRE multi-site datasets in Alzheimer’s Disease and schizophrenia, respectively, have both been used in “challenges” open to all comers who have data mining techniques to identify who has the disease and who doesn’t, in support of new diagnostic techniques [51, 52]. The original study designers and data collectors for any given project cannot have all possible analysis and statistical techniques at their fingertips; therefore, these data repositories are immensely valuable as ongoing resources for the research community.

While the idea of a large and representative dataset is appealing, a challenge with data collected over multiple imaging sites is the variability in the resulting images that is not due to subject differences, but simply due to the scanner and imaging parameters—i.e., increased noise that could swamp more subtle disease-specific effects. Planned studies with tightly controlled protocols minimize this variability, giving the best chance for identifying smaller individual differences [53]. A good example is the ADNI study previously mentioned, a large and carefully planned multi-site study of subjects with Alzheimer’s Disease (AD), subjects with Mild Cognitive Impairment, and healthy controls. Their methods have allowed them to identify clusters of pre-diagnosed subjects with different prognoses, some of whom are more likely to convert to full AD than are others [54].

Studies with less controlled designs must work with the data they can access, which entails only identifying variables with effects that are robust to the sources of imaging or clinical data collection heterogeneity. In combining common variables across legacy data, the more opportunistic studies often cannot benefit from the deep phenotyping that can make analyses like ADNI’s more rich. However, planned studies often do not collect broadly useful measures either, as noted previously. They focus on the hypotheses they were funded to study, and often do not have additional information about the subjects that would make the data re-usable for another question; in contrast, institutional approaches can leverage that breadth. Through minimal standard imaging protocols and planned data sharing approaches, datasets with consistent imaging methods and a wide array of clinical measures can be potentially aggregated for data mining.

The rise of these large scale studies, hand-in-hand with the recognized emphasis on sharing the resulting data, has also provided numerous data repositories and an increased awareness of the data’s value [55, 56]. MRI data repositories that are open to the research community are funded by the National Institutes for Health (NIH), individual institutions, or individual laboratories (for example [57–61]). However, the current efforts in data sharing are often hampered by the lack of standardization not only in what is collected, but also how it is described. Data integration and mediation is an ongoing challenge that is a large part of the field of neuroinformatics (see e.g., [62–66]). The data are not necessarily compatible when combined across different sources, with many missing or questionable data points.

A primary challenge, besides the noisiness of the data collection methods and the ability to find datasets others have already collected, is the science of working with”big data”. What questions can be asked given the data that has already been collected and made available? Given one’s scientific question; could the hypothesis be tested in available data, rather than designing a new study from scratch? How does one handle the noise, uncertainty and missing data? This requires the next generation of neuropsychiatric researchers to understand that these big datasets exist; how to use the neuroinformatics tools and methods to find them, as well as the best practices for aggregating the data or performing meta-analyses while addressing the inescapable sources of variance.

## Conclusions

Large-scale neuroimaging studies of varying designs have been increasingly applied to neuropsychiatric research. The studies vary from completely controlled data collection and analysis, to *post hoc* meta-analyses with no control over those experimental parameters. Each category of experimental design has its strengths and weaknesses in its ability to address sources of variation, and its ability to identify subtle effects of interest.

Successful data integration and mediation will make the re-use of these datasets more viable and valuable. An imaging dataset of 20 subjects can provide a few findings, but an underpowered study has an increased risk of inflating its estimates of effect size, leading to a lack of reproducibility [67]. But, in conjunction with 10 or 100 more studies of similar size and type, it can reliably help address questions of clinical importance about symptom variations, prognosis or genetic influences. There were 12,000 papers published in English in 2012 as found in PubMed using the query “((human brain mapping) OR (fMRI) AND (brain AND MRI)”. Even if only one-third of them represent unique imaging datasets, there are clearly a plethora of imaging datasets of the human brain in various states that could be shared, reused or aggregated for novel analyses.

Training in experimental psychology and cognitive neuroscience often focuses on the details of experimental design for *de novo* data collection and analysis. However, while good experimental design is key, *de novo* data collection need not be. Neuroimaging researchers need to take a page from the sciences of climatology and geology, from economists and others who cannot always manipulate the environment in a precisely controlled manner to test their models. We are now at a point in the neuroimaging domain where neuroimaging researchers should first ask whether their question can be refined or even answered in the agglomeration of data previous researchers have collected. An even stronger approach would be to consider, when collecting new data, not only how to use existing data to supplement the proposed data collection, but how the new data could be used by others in the future, and how best to design the experiments and resource allocation for the project to facilitate that re-use. This is, in effect, combining computational and semantic web methods with statistical methods, for a “big data” approach to available neuroimaging data.

## Author information

Dr. Turner has been working with MRI studies since 1998, and with multi-site imaging of schizophrenia since joining the FBIRN study in 2003 as the project manager, as well as participating in the MCIC and COBRE studies, the first phase of ADNI, and other multi-site clinical imaging studies. Her research encompasses brain correlates of different psychological states, and the genetic influences underlying schizophrenia in particular. She is committed to neuroimaging data sharing, developing the Cognitive Paradigm Ontology, chairing the ENIGMA Schizophrenia Working Group, and participating in the International Neuroinformatics Coordinating Facility’s Neuroimaging Data Sharing Task Force. She is currently an Associate Professor in the Department of Psychology and Neuroscience Institute at Georgia State University, Atlanta.

## Abbreviations

ABIDE: Autism brain imaging data exchange
AD: Alzheimer’s disease
ADNI: Alzheimer’s disease neuroimaging initiative
BIG: Brain imaging genetics project
COBRE: Center of Biomedical Research Excellence
CORR: Consortium for Reliability and Reproducibility
CT: Computed tomography
ENIGMA: Enhancing Neuro Imaging Genetics through Meta Analysis
FBIRN: Functional Biomedical Informatics Research Network
fMRI: Functional magnetic resonance imaging
GOBS: Genetics of brain structure
MCIC: Multi-site Clinical Imaging ConsortiumNIH: National Institutes of Health
TBI: Traumatic brain injury
TOP: Thematically Organized Psychosis.
